# Transforming GP practices into multiprofessional primary care centers: The role of nursing professionals (PRIMA) – study protocol for a multicenter non-randomized, controlled, parallel-group intervention study

**DOI:** 10.1371/journal.pone.0357117

**Published:** 2026-09-09

**Authors:** Philipp Naß, Wiebke Schüttig, Amelie Flothow, Marie Coors, Leonie Sundmacher, Regina Stolz, Anika Klein, Stefanie Joos, Iris an der Heiden, Ann-Kathrin Jöchner, Hans-Dieter Nolting, Hannah Haumann, on behalf of the PRIMA Study Group

**Affiliations:** 1 Department of Health Economics, Technical University of Munich, Munich, Germany; 2 Institute of General Practice and Interprofessional Care, University Hospital Tuebingen, Tuebingen, Germany; 3 Institute for Health and Social Research Berlin, Germany; PLOS: Public Library of Science, UNITED STATES OF AMERICA

## Abstract

**Background:**

Germany faces increasing challenges in primary care due to demographic change, workforce shortages, and the growing prevalence of chronic conditions. Integrating nursing professionals into primary care teams may improve continuity, comprehensiveness, and efficiency of care, but evidence for the German setting remains limited. The PRIMA project (DRKS00039104) implements a new care model that transforms general practices into multiprofessional primary care centers integrating specially trained nursing professionals. The objective is a productivity- and quality-oriented reorganization of care for patients with chronic diseases. The intervention targets patients with high-risk chronic diseases. Core elements include extended nursing services, subsidiary task allocation within the practice team, structured organizational development, and optional use of a medical triage software.

**Methods:**

Effectiveness and health-economic outcomes are evaluated in a prospective, quasi-experimental, controlled study under real-world conditions. The intervention group includes patients from 20 participating practices; the control group is matched using routine claims data. The primary endpoint is the number of ambulatory care–sensitive emergency cases after 12 months, analyzed intention-to-treat. Secondary outcomes include healthcare utilization, mortality, continuity, and coordination of care. Statistical analyses will account for confounding and clustering at practice and patient level. The health economic evaluation will estimate incremental cost-effectiveness ratios based on ambulatory and total healthcare costs. A parallel process evaluation uses longitudinal mixed-methods. It comprises interviews, activity logs and focus groups, and surveys among patients, their relatives, practice teams and collaborators. Key aspects assessed include implementation facilitators and barriers, organizational development and interprofessional collaboration, nurse role development, and patient quality of life and treatment satisfaction.

**Discussion:**

Findings are highly relevant for strengthening primary care in Germany and internationally, where multidisciplinary primary care centers are increasingly promoted. If successful, the intervention could reduce emergencies and improve patient outcomes, serving as a scalable model and informing policy and reimbursement strategies.

## Introduction

The provision of primary care by general practitioners (GPs) and their teams in Germany is already at risk in many regions and is expected to become a central challenge over the next two decades due to demographic change and decreasing workforce in this care sector [1,2]. The consequences affect both the individual patient – whose care may no longer be adequately ensured – and the healthcare system as a whole – as inappropriate utilization of specialist care, emergency departments, and avoidable hospitalizations are likely to increase.

Multiple strategies targeting different levels of the healthcare system, including medical education, postgraduate training, and incentives for practice establishment, have been introduced to strengthen the workforce within primary care. Nevertheless, demographic developments, increasing complexity of care, and limited physician availability suggest that expanding the physician workforce alone will not adequately address future primary care needs [3]. This particularly concerns continuity of care, which is essential for patients with chronic conditions and multimorbidity, as well as increasing needs related to counselling, prevention, and self-management support [4]. Strengthening organizational structures and workflows within primary care practices is necessary to ensure that limited physician capacity is reserved for activities requiring medical decision-making and clinical expertise. Tasks that do not require physician qualification may be delegated to adequately trained non-physician personnel or streamlined through process redesign and technological solutions. One internationally established approach is the integration of (academically trained) nursing professionals into primary care teams [5–7].

Traditionally, German primary care teams consist of general practitioners and medical assistants (“Medizinische Fachangestellte”). While nurses, particularly Advanced Practice Nurses (APNs) are well established in primary care internationally, they have so far only played a marginal role within German general practice teams. Unlike many international healthcare systems, nursing in Germany has traditionally been rooted in non-academic vocational training, and the academic institutionalization of nursing science is still evolving. Furthermore, legal, professional, and reimbursement regulations governing the integration of academically trained nurses into primary care settings remain fragmented and incompletely defined. This has mainly been attributed to three structural factors: (1) a traditionally physician-centered healthcare system based on delegation rather than substitution of medical tasks, (2) a predominantly physician-oriented reimbursement system in ambulatory care, and (3) historically developed professional structures in which physician practices are primarily staffed by medical assistants. In recent years, expanded qualification pathways for non-physician practice staff, including VERAH (“Versorgungsassistentin in der Hausarztpraxis”, GP-Practice Assistant), NäPA (“Nichtärztliche Praxisassistenz”, Non-medical Practice Assistant), physician assistants and academic programs such as Primary Care Management, have contributed to changes in the composition and scope of German general practice teams. Nevertheless, nurses, and their profession-specific competencies continue to play a minor role within German general practice teams, even when employed in practices, where they frequently work in roles comparable to those of medical assistants. Evidence from primary care settings suggests that the involvement of APNs is associated with outcomes comparable or superior to traditional physician-led care regarding safety, clinical outcomes, costs, and patient satisfaction, although resource utilization may differ between models of care [6,8–13]. Heterogeneous findings regarding efficiency and costs have partly been attributed to longer consultation times and more frequent follow-up visits, which may nevertheless contribute to improved disease management and reduced hospitalization rates [7,10]. Empirical evidence regarding the integration of nurses in general, and APNs specifically, into German general practice teams remains limited. Nevertheless, recent third-party funding projects have indicated that the inclusion of academically trained nurses in primary care teams may improve quality of care, patient-centeredness, and relieve physician workload [14,15]. While patients generally report high acceptance of transferring selected care tasks to nurses, several barriers remain, including unclear legal frameworks, insufficient role clarity, and unresolved reimbursement structures [6,16].

Against this background, the PRIMA project aims to implement a novel model of care by transforming general practices into multiprofessional primary care centers (PRIMA), in which nurses, either academic or non-academic and in the following referred to as “PRIMA nurse” (PN), are integrated into general practice teams. This model of care utilizes the inherent competencies of an experienced nurse to enhance continuity, comprehensiveness and subsidiarity of care and aims at enabling an efficient allocation of tasks according to professional competencies present in the practice team (e.g., medical, administrative and nursing competencies). Within this care model, activities of the PN are classified into seven predefined interventions for patients with chronic conditions that extend beyond routine care and were developed based on the Chronic Care Model (CCM) [17]. Based on this, a remuneration system is introduced that supports the sustainable implementation of these structural and process changes. PRIMA targets patients with chronic conditions, especially those at high risk of decompensation or signs of instability. As demographic change and the increasing prevalence of chronic diseases pose significant challenges to care delivery, these patients have been shown to benefit from nurse-integrated primary care teams [18].

The study evaluates the effectiveness, implementation, and economic implications of the new care model (NCM) at micro-, meso-, and macro-levels. At the micro-level (patient level), the study examines whether the NCM improves predefined primary and secondary outcomes compared with usual care. It is hypothesized that the intervention supports appropriate treatment, facilitates early identification and management of deterioration and complications, strengthens patient self-management and caregiver involvement, and thereby reduces ambulatory care-sensitive emergency cases (ASE) while improving patient-relevant outcomes. In addition, patient and caregiver perspectives on the intervention will be assessed.

At the meso-level (practice level), the study investigates whether the NCM can be implemented as intended. Furthermore, the implementation process, including facilitators and barriers to implementation as well as effects on workflows and interprofessional collaboration will be evaluated.

At the macro-level (health system level), the study assesses the costs and cost-effectiveness of the NCM from the perspectives of healthcare payers and participating primary care practices. It is hypothesized that the intervention may reduce healthcare expenditures through fewer ASE and more efficient utilization of healthcare services, while changes in practice organization and reimbursement structures may also generate economic benefits for participating practices.

## Materials and methods

### Patient and public involvement

Stakeholder involvement was incorporated throughout the development and implementation of the project. During the conceptual phase, two advisory meetings were conducted with a physician advisory board that included representatives of the German Association of General Practitioners (Hausärztinnen- und Hausärzteverband) and MEDI, a large interdisciplinary network representing office-based physicians and psychotherapists in Germany. During the implementation phase, the project is supported by a scientific advisory board comprising representatives of the German Nurses Association (DBfK), nursing scientists, an APN, a patient representative from LAG-Selbsthilfe, a regional umbrella organization for patient self-help groups, a representative of the German Association of General Practitioners, and a GP working in a primary care unit in Austria. The advisory board meets annually and provides independent advice to the project team regarding the implementation and evaluation of the intervention.

### Aim

The aim of PRIMA is to develop primary care teams in general practices into multiprofessional primary care teams by integrating PNs with specific tasks. Based on the CCM, a NCM optimizing the management of patients with chronic conditions will be implemented [17].

### Design

The PRIMA study is a non-randomized, controlled, cluster-based, quasi-experimental intervention study. The study is designed as a superiority trial, aiming to demonstrate improved outcomes of a multiprofessional primary care model compared to usual care. The intervention group (IG) consists of the patients recruited from the 20 participating GP practices. The control group (CG) will be established through matching based on routine data. In addition to the effectiveness evaluation, a mixed-methods process evaluation (PE) based on the Consolidated Framework for Implementation Research (CFIR) will be conducted to assess implementation processes, contextual factors, and intervention fidelity [19]. The PE includes team members from the participating practices, participating patients and their relatives as well as collaborators. For both quantitative and qualitative data, a longitudinal approach is applied, the latter reflected in qualitative case studies encompassing interviews, qualitative activity logs and focus groups. Repeated data collection will enable the investigation of evolving implementation processes, barriers, and facilitators across different stages of implementation and give insight in the implementation, adaption and integration of the intervention into routine practice over time.

### Setting

The trial is conducted in the German ambulatory primary care setting, namely in 20 general practices located in the federal state of Baden-Württemberg, Germany. The participating practices provide routine outpatient care services within the German statutory health insurance (SHI) system.

The target population of PRIMA are patients with a chronic condition as defined within the German statutory healthcare system. Within this system, a chronic condition can be identified on the Uniform Value Scale (EBM), which is jointly developed by the National Association of SHI Physicians and the National Association of SHI Funds. The conceptual basis of this definition is derived from German social law, in particular the definition of “severe chronic disease” according to § 62 of the Social Code Book V, and its specification by the Federal Joint Committee [20]. Within the EBM framework, chronic illness is operationalized for billing purposes in the billing code chronic care reimbursement (“Chronikerpauschale”: 03220/03221) [21]. Patients are classified as chronically ill if they have a documented, continuous need for medical care due to the same confirmed diagnosis over a period of at least 12 months. This requirement is typically operationalized through repeated physician–patient contacts across multiple quarters rather than pre-defined clinical criteria, usually in at least three out of four consecutive quarters, reflecting sustained utilization of care. In addition, an ongoing need for treatment must be present. Thus, the EBM-based definition represents an administrative and utilization-based proxy for chronic illness, grounded in legal and regulatory frameworks rather than purely epidemiological or clinical definitions. Patients eligible for inclusion in the study were identified from the population of individuals receiving the chronic care reimbursement billing code (“Chronikerpauschale”), which served as the source population for the study. Additional clinical eligibility criteria were subsequently applied to identify patients with an increased risk of care instability or decompensation in a step-wise approach.

### Sample size calculation

For the sample size and power calculation, a baseline rate of 0.20 ASE per patient per year is assumed. In the general population, ASE rates range between 4% and 8% [22]. As the study population consists of patients with chronic conditions selected based on clinical judgement and expected to benefit from enhanced care, a higher baseline rate is expected. Based on published evidence suggesting a 20–40% reduction in ASEs through improved ambulatory care [23], a conservative effect size of 15% is applied.

The study follows a parallel cluster design with 20 practices per arm and approximately 112 patients per practice. This accounts for an anticipated dropout of 20% at practice level and 10% at patient level. The design includes 12 months of pre- and 12 months of post-intervention observation [24]. An intra-cluster correlation coefficient (ICC) of 0.01 is used. Under these assumptions and a two-sided alpha of 5%, the study achieves a statistical power of 82% to detect a 15% reduction in ASE rates.

The PE within PRIMA does not assess a primary endpoint of the study. Therefore, no separate sample size calculation was required for the PE. Instead, the sample size of the PE is determined by the total population of all participant groups included in the PRIMA study and by the methodological approach chosen within the PE sub-projects. For the questionnaire-based quantitative PE, all patients included in the main study as well as all staff members of the participating practices will be included (estimated n = 2,000). To ensure sufficient thematic breadth regarding the research questions – while simultaneously aiming for thematic saturation – a total of 4 practices will be included in the qualitative case studies, leading to about 60 individual interviews (30–60 minutes) with patients, relatives, and practice staff, along with approximately 16 qualitative activity logs. In addition, 4–5 cross-site focus groups (approximately 120 minutes each) with members of the PRIMA teams and stakeholders at interface levels (e.g., care support centers) are planned.

### Inclusion and exclusion criteria

The predefined inclusion and exclusion criteria used to determine study eligibility are summarized in Table 1.

**Table 1:** Inclusion and exclusion criteria.

	Inclusion criteria	Exclusion criteria
Patient Level	• Age ≥ 18 years • At least one life-limiting chronic condition reflected by at least two billings of the German chronic care fee (“Chronikerpauschale”, EBM GOP 03220/03221 or equivalent under selective contract) in two of the four quarters preceding study enrolment • Continuous treatment in the participating practice for at least four quarters prior to inclusion • Patients in an unstable phase of chronic disease with ASE related to their chronic condition and/or hospitalizations due to their chronic condition within the past three years • Patients in a stable phase of chronic disease with at least one sign of a high risk of decompensation*	• Estimated life expectancy based on the assessment of the treating GP at the start of the intervention of less than one year (minimum intervention period)
Practice Level	• Practices providing routine care as GPs in the federal state of Baden-Württemberg, Germany • Employment of a nursing professional	• Participation in similar studies

*The risk of decompensation was based on the clinical judgement of the participating GPs in regard of the following aspects: presence of multimorbidity with geriatric characteristics (e.g., immobility, cognitive impairment, or frailty), unfavorable living circumstances (e.g., living alone, partner requiring care, absence of nearby relatives), long-term care dependency (Pflegegrad, care level), frequent changes/dose adaptions in medication, increasing frequency of acute exacerbations or clinically relevant deterioration in vital parameters (e.g., blood pressure, pulse, oxygen saturation, change in weight) indicating disease progression, and reduced ability to participate in or adhere to the care processes.

### Recruitment

#### Intervention group – participating practices, PNs and patients.

Participating practices were recruited between January and December 2025, while information about the PRIMA project had already been shared in the two months leading up to the project start. Recruitment was conducted by the Association of SHI Physicians of Baden-Württemberg (KVBW) through two sequential steps. First, postal letters were sent to a sample of randomly selected practices, followed by information and outreach via email to a further randomly selected sample of practices in the federal state of Baden-Württemberg, Germany. PNs employed in PRIMA were recruited by the practices themselves or via the KVBW. All PNs participating in PRIMA must have completed a three-year vocational training program as nursing professional (*Altenpfleger:in*, *Gesundheits- und Krankenpfleger:in* or *Pflegefachfrau/-mann*) and possess either an academic postgraduate qualification in nursing or at least five years of professional experience in nursing, preferably across different care settings. Alternatively, nurses who have obtained an academic qualification in nursing, such as a bachelor’s or master’s degree with a specialization in Advanced Nursing Practice could be employed. Patients eligible for participation were identified in a stepwise approach using a combined data-driven approach and clinical judgement by the GP and the respective practice team based on the inclusion and exclusion criteria. All study participants are recruited between 1 January 2026 and 30 September 2026. Eligible patients are informed about the study during routine consultations. Written informed consent is obtained by the treating GP and forwarded to KVBW prior to participation using a standardized consent form approved by the ethics committee. Where applicable, consent must be provided by a legal guardian or authorized representative.

For the PE, all study participants with the capacity to provide informed consent are invited by the PN to participate in the surveys of the PE. Recruitment of the practice teams, patients as well as their relatives and collaborators for the qualitative case studies is guided by the scientists from Institute of General Practice and Interprofessional Care, Tuebingen (IAIV) in a selective sampling approach guided by the principle of information power. Informed consent was obtained from all participants in the PE [25].

### Control group – patients and practices

The CG consists of matched patients receiving usual care in comparable general practices outside the PRIMA intervention. Patients will be identified using routine data from the KVBW and AOK Baden-Württemberg (AOK BW) databases and matched to participants in the IG at both patient and practice level. Matching criteria include demographic and clinical characteristics such as age, sex, diagnosis, and comorbidities to ensure comparability between groups. Usual care reflects standard ambulatory primary care within the German statutory healthcare system as delivered by GPs outside the PRIMA intervention. This allows a pragmatic and policy-relevant evaluation of the intervention under real-world conditions, assessing the added value of the PRIMA care model compared to standard practice without altering routine care pathways. There is no control group for the PE.

### Study timeline

**Fig 1:**
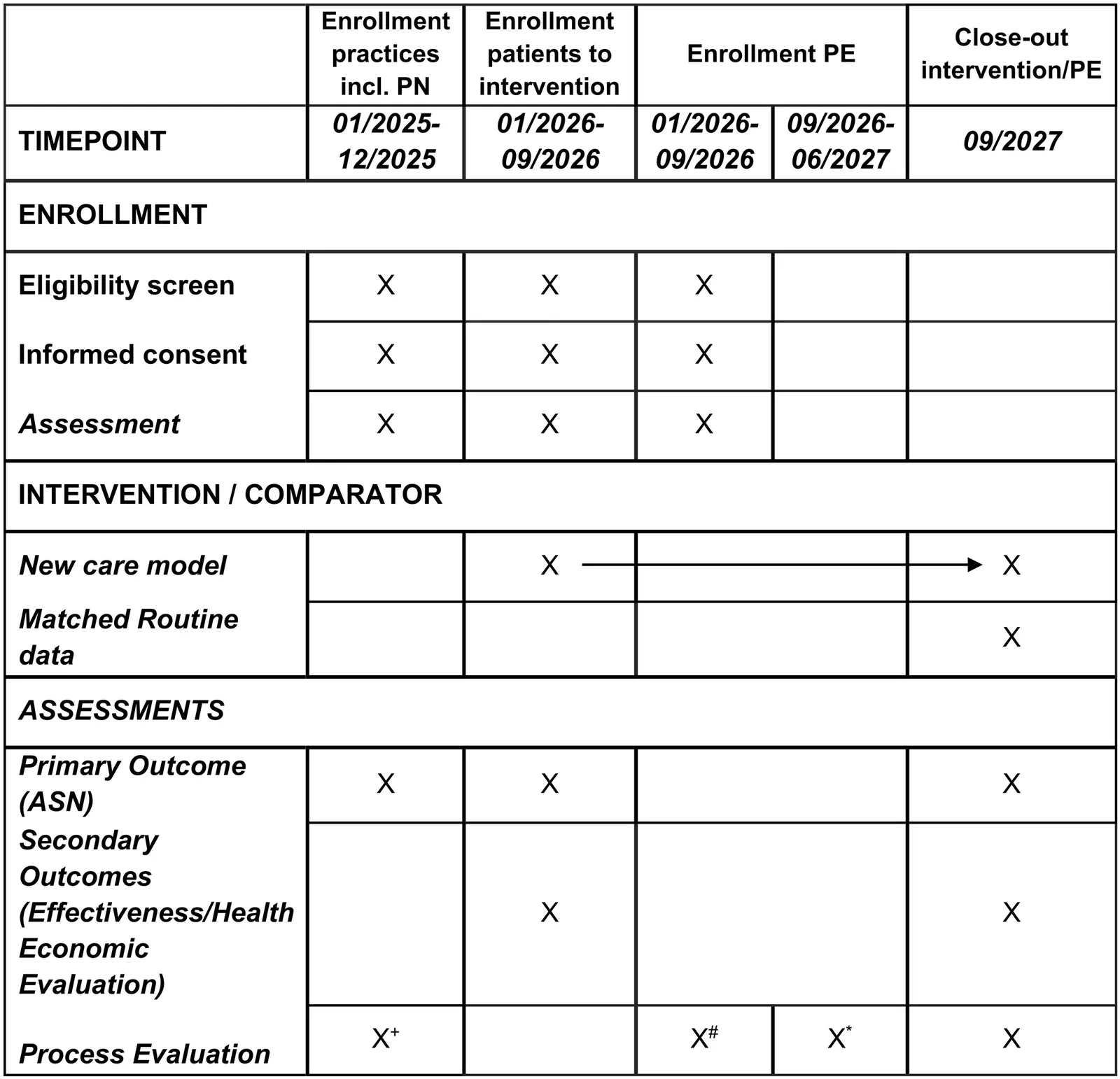
Participant timeline: Schedule of enrollment, interventions, and assessments [64]. + This time point reflects enrollment of practice teams to quantitative PE. # This time point reflects enrollment of patients to quantitative PE and practice teams to qualitative case studies. *This time point reflects enrollment of patients/relatives/collaborators to the qualitative case studies of PE.

The study consists of a preparation phase, an intervention phase, and an evaluation phase (Fig 1). Preparatory activities, including practice recruitment, development of study materials, training, and establishment of data management procedures, started in January 2025 and continued until December 2025. The intervention phase is scheduled from January 2026 to September 2027 and includes patient recruitment for the intervention and PE as well as the implementation of the intervention. This study was registered retrospectively on 26 April 2026 (DRKS00039104) after enrolment began, due to administrative delays during the project start-up phase. No changes to the objectives, eligibility criteria, endpoints, or statistical analysis plan were made after enrolment began. The authors confirm that all ongoing and related trials for this intervention are registered. At the time of manuscript submission, recruitment of participating patients is ongoing and expected to be completed in September 2026. Data collection will be completed in September 2027. The evaluation and analysis phase will take place between October 2027 and June 2028 and will include data analysis, reporting, and dissemination of study findings. Study results are expected in 2028.

### The PRIMA intervention

**Fig 2:**
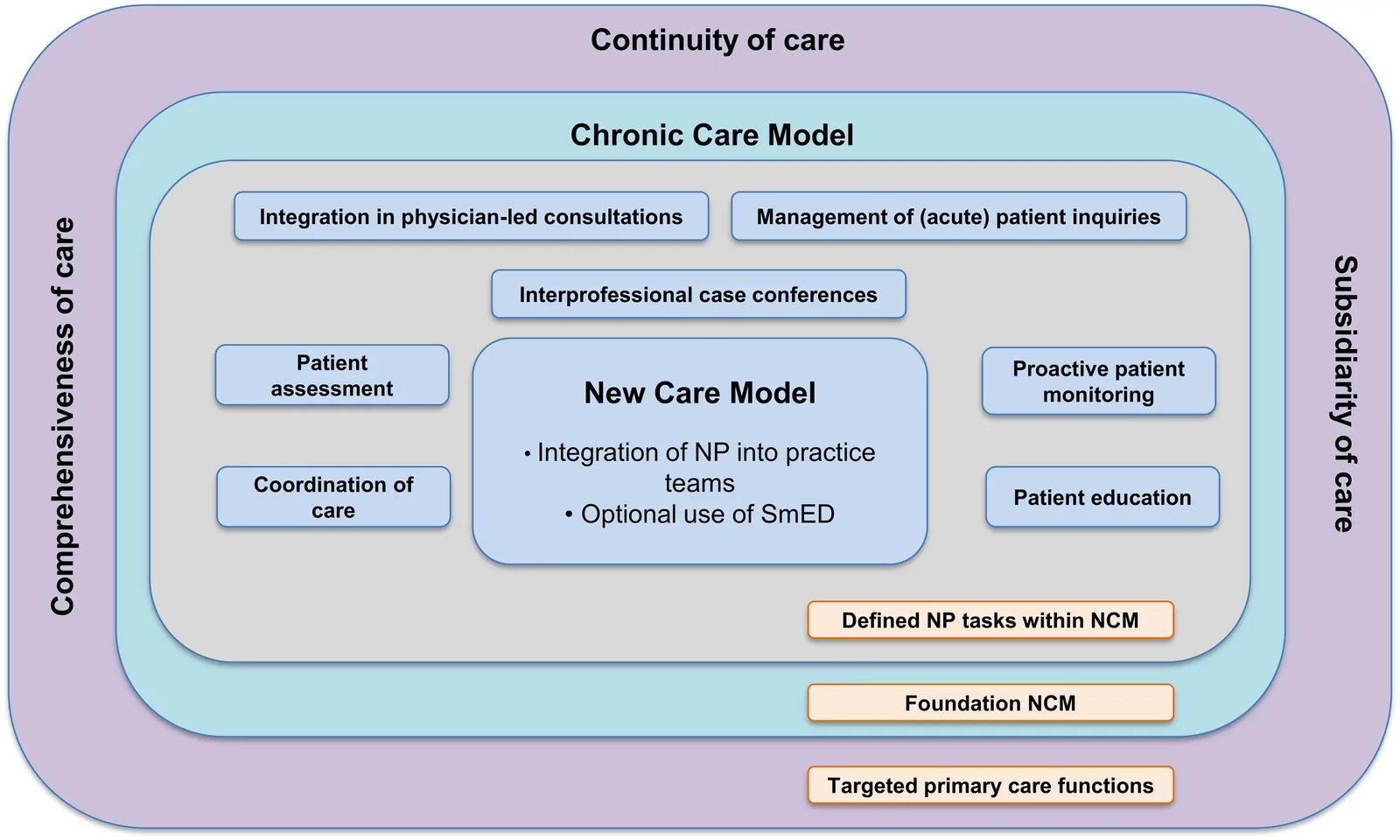
New care model in PRIMA.

The PRIMA intervention encompasses different elements at the practice level focusing on the aspects of continuity, comprehensiveness and subsidiarity of care provided by the practices (Fig 2). Core of the intervention is the introduction of PNs with their profession-specific competencies to the existing practice team. Intervention development was guided by the CCM. The PN introduces a NCM offering services beyond regular care currently provided. Within this NCM, the PN is to deliver seven defined tasks in the care for patients with chronic conditions, namely: 1. integration of PN into regular physician-led consultations, 2. conduct of initial and follow-up assessments to support comprehensive evaluation of care in the home setting, 3. coordination of care, 4. participation in interprofessional case conferences, 5. proactive monitoring of the care trajectory, 6. provision of patient education and 7. management of patient inquiries, including acute intervention in crisis. The intervention will be delivered over the full observation period, with a minimum duration of 12 months of follow-up per patient. In order to prepare the intervention, the PNs were employed six months in advance to the start of the intervention in January 2026. Three workshops of each two full days were held before the start of the intervention to prepare the PNs for the NCM. The workshops encompassed information on the PRIMA study and its design, including information and training for the seven tasks to be delivered. Furthermore, the workshop included topics like working in multiprofessional teams, communication skills and the own understanding of the PN role within the practice team. Prior to the start of the intervention, GPs and other team members were invited to participate in two so called “network meetings” providing the participating practice teams as a whole with relevant information regarding the conduct of the study. During the intervention phase, quality circles for the PNs take place on a monthly basis to support intervention delivery. Furthermore, during the first six months of the intervention, another two-day workshop will be held. As the role of the PN with defined tasks beyond regular care is novel to the practice teams, change management support is offered. All PN patient contacts are documented by the PN in a work diary during the intervention phase. Documentation includes baseline data (e.g., age, sex, date of inclusion, presence of inclusion criteria) and reason for contact, type of contact, activities performed, provided healthcare services, time expenditure (minutes), and, where applicable, additional comments in a free-text field. All elements of the intervention are delivered by the project partner IGES in collaboration with KVBW.

Participating practices are offered the optional use of SmED (Strukturierte medizinische Ersteinschätzung in Deutschland, structured medical initial assessment tool), a structured software tool for medical initial assessment. SmED supports urgency assessment and facilitates allocation of patients to the most appropriate professional within the practice team.

Participants in the PRIMA study may receive any concomitant care as part of routine healthcare within the German statutory healthcare system. The intervention is delivered as part of routine primary care in participating practices and does not interfere with or restrict other aspects of patients’ healthcare utilization.

As the PRIMA intervention is an organizational care model implemented at practice level, adherence is primarily supported through structured training of participating practice teams, and PNs especially, provision of implementation support, and continuous contact with the study coordination team. No specific adherence measures are applicable for comparator patients, as the CG is based on routine data reflecting usual care without active participation requirements.

Participants will remain in the analysis population unless they withdraw consent or data availability ceases. In case of death, participants are retained in the intention-to-treat (ITT) population, and data are included up to the time of death. As the PRIMA intervention is implemented at the practice level as an organizational care model, no individual-level modification of the intervention is foreseen. Practices may discontinue participation in the intervention in case of organizational changes. However, no modifications of the intervention at individual patient level are planned.

### Outcomes

The primary outcome of PRIMA is the number of ASE after 12 months, identified using routine data from SHI physicians via standardized reimbursement codes (GOP). ASEs are defined as acute emergency contacts that are considered potentially avoidable through effective outpatient care and adequate disease management [22].

Secondary outcomes include measures of healthcare utilization, continuity and coordination of care, preventive service delivery, mortality data and economic outcomes. These are derived from routine data provided by KVBW and AOK BW.

Outcomes of the health economic evaluation include the costs of ambulatory care, defined as cumulative ambulatory care costs derived from claims data over a 12-month follow-up period, as well as intervention costs incurred during this period (including costs related to the PN intervention). In addition, cost-effectiveness will be assessed by relating total costs to the number of ASE.

Outcomes are assessed either over 12 months or across the full intervention period and obtained from routinely collected claims data made available by the KVBW and AOK BW following completion of the observation period. They are listed in Table 2 together with their respective data sources and assessment timeframes.

**Table 2:** Overview of study outcomes, data sources, and measurement periods.

Primary outcome				
Level	Outcome	Definition	Data source	Timeframe
Micro	Ambulatory care–sensitive emergencies (ASEs)	Number of ASEs identified via billing codes (GOP 01210 and 01212); indicator of potentially avoidable emergency care	KVBW routine data	12 months
**Secondary outcomes – KVBW data**				
Micro	ASE per person-year	ASE rate adjusted for patient-specific observation time	KVBW	Intervention period
Micro	Patients with ≥1 ASE	Proportion of patients with at least one ASE	KVBW	12 months
Micro	Ambulatory emergencies	All emergency contacts independent of diagnosis	KVBW	12 months
Micro	Ambulatory emergencies per person-year	Rate-adjusted emergency contacts	KVBW	Intervention period
Micro	GP utilization	Number of GP contacts / quarters with GP visits	KVBW	Intervention period
Micro	Specialist utilization	Number of specialist contacts / quarters	KVBW	Intervention period
Meso	Treatment intensity	EBM points per patient and per practice	KVBW	Intervention period
Meso	Continuity of care	Continuity-of-Care Index (e.g., Bice–Boxerman)	KVBW	Intervention period
Meso	Care coordination	Number of providers; referral-based specialist visits	KVBW	Intervention period
Micro	Preventive care	Vaccination rates (influenza, pneumococcal, COVID-19)	KVBW	Intervention period
Macro	Outpatient costs	Cumulative outpatient costs incl. intervention costs	KVBW	12 months
Macro	Outpatient cost-effectiveness	Cost per outcome achieved	KVBW	12 months
**Secondary outcomes – AOK Baden-Württemberg data**				
Macro	ASE + ambulatory-sensitive hospitalizations	Combined outpatient and inpatient avoidable emergency events	AOK BW	12 months
Macro	ASE + hospitalizations per person-year	Rate-adjusted combined events	AOK BW	Intervention period
Macro	Total emergencies	Diagnosis-independent emergency contacts	AOK BW	12 months
Macro	Total emergencies per person-year	Rate-adjusted total emergencies	AOK BW	Intervention period
Macro	Ambulatory-sensitive hospitalizations (ASH)	Potentially avoidable hospital admissions	AOK BW	12 months
Macro	ASH per person-year	Rate of ASH	AOK BW	Intervention period
Macro	Overall survival	Mortality data	AOK BW	Intervention period
Macro	Guideline adherence	Medication prescribing patterns	AOK BW	Intervention period
Macro	Total healthcare costs	All-cause expenditure	AOK BW	12 months
Macro	Overall cost-effectiveness	System-level cost-effectiveness	AOK BW	12 months

The PE encompasses outcomes on the micro- and the meso-level, namely participating patients and their relatives as well as the practice teams. The (PE) evaluation comprises both quantitative and qualitative measures. Table 3 gives an overview of the outcomes. All validated questionnaires have been used in primary care settings, targeting either interprofessional teams or patients with chronic conditions, aligning with the objectives of this study. Self-developed questionnaires were piloted with representatives of the target population using the think-aloud method to assess comprehensibility, relevance, and usability. Findings from the pilot testing informed revisions of the questionnaires before their use in the study [26].

**Table 3:** Outcomes of process evaluation on Meso and Micro Level.

Meso Level (Practice Teams)						
Outcomes	Instrument	B	T0	T1	T2	P
Job satisfaction	Warr-Cook-Wall (WCW) [40–42]	x		–	X	PT
Interprofessional Collaboration	Assessment of Interprofessional Team Collaboration Scale (AITCS-II) [7,43,44]	x		–	X	PT
Interprofessional Socialization	Interprofessional Socialization and Valuing Scale (ISVS 9a + 9b) [45,46]	x		–	x	PT
Implementation fidelity	Self-developed Questionnaire	–	–	–	x	PT
	PN documentation (activity logs)				x	PN
	Qualitative Case Study (interviews, focus groups, qualitative activity logs)			x	x	PT/C
PN training	Self-developed questionnaire based on ICAP model and Kirkpatrick levels [47–49]		x		x	PN
Implementation Outcomes Barriers & Facilitators Role Transformation	Qualitative Case Study (interviews, focus groups, qualitative activity logs)		x	x	x	PT/C
Practice Characteristics	Self-developed questionnaire	x				
**Micro Level (patients and their relatives)**						
Quality of life	EQ-5D-5L [50–53]	–	X	–	x	P
Self-Efficacy	Self-Efficacy-Scale (SES6G) [54]	–	X	–	x	P
Subjective symptoms and treatment satisfaction	Measure Yourself Medical Outcome Profile (MYMOP) [55,56]	–	X	–	x	P
Satisfaction with care by PN	Nurse Practitioner Satisfaction Survey (NPSS) [57,58]	–	–	–	x	P
Patients assessment of chronic illness care	PACIC short [59–63]	–	x		x	P
Patients’ experiences of the new model of care	Qualitative Case Study	–	–	–	x	P/R

B: Baseline, Start of NP in Practice Teams, T0: Start of Intervention, T1: Mid Intervention, T2: End of Intervention; ICAP: Interactive –Constructive-Active-Passive; P: Population, PT: Practice Team; PN: Prima nurse, C: collaborators; R: relatives

### Data

Regarding the effectiveness analysis and economic evaluation, routinely collected healthcare data will be used. Data sources include routine data from KVBW and AOK BW for the reporting period from 01 January 2024–30 September 2027. KVBW includes patient core data, information on participation in the GP-centered care model collected via the study consent form, practice core data, regional indicators, and outpatient claims data. For the subset of AOK BW-insured patients, patient master data, data on outpatient and inpatient care, pharmaceutical prescriptions, remedies and medical aids, long-term care, rehabilitation, sick leave, transportation costs, and domestic help are provided by AOK BW.

Written informed consent will be obtained from all patients participating in the IG prior to inclusion in the study and prior to the transfer and use of routinely collected healthcare data for research purposes. Only routinely collected healthcare data necessary for the predefined analyses will be requested and processed in accordance with the principle of data minimization. KVBW data will be provided as separate datasets for intervention and control groups. A matched CG will be generated from KVBW data based on criteria defined by the evaluator. AOK BW data will be provided as a pre-matched dataset, with matching performed by AOK BW prior to data delivery. Datasets from KVBW and AOK BW will not be linked or merged, as all outcomes are pre-specified to be analyzed within their respective datasets.

Data extraction follows routinely implemented administrative processes and is performed on a quarterly and continuous basis. Data completeness, validity and quality are ensured by standard validation procedures at the respective data providers, and additional plausibility and validity checks are performed at the Technical University of Munich (TUM) data trust office prior to analysis.

**Fig 3:**
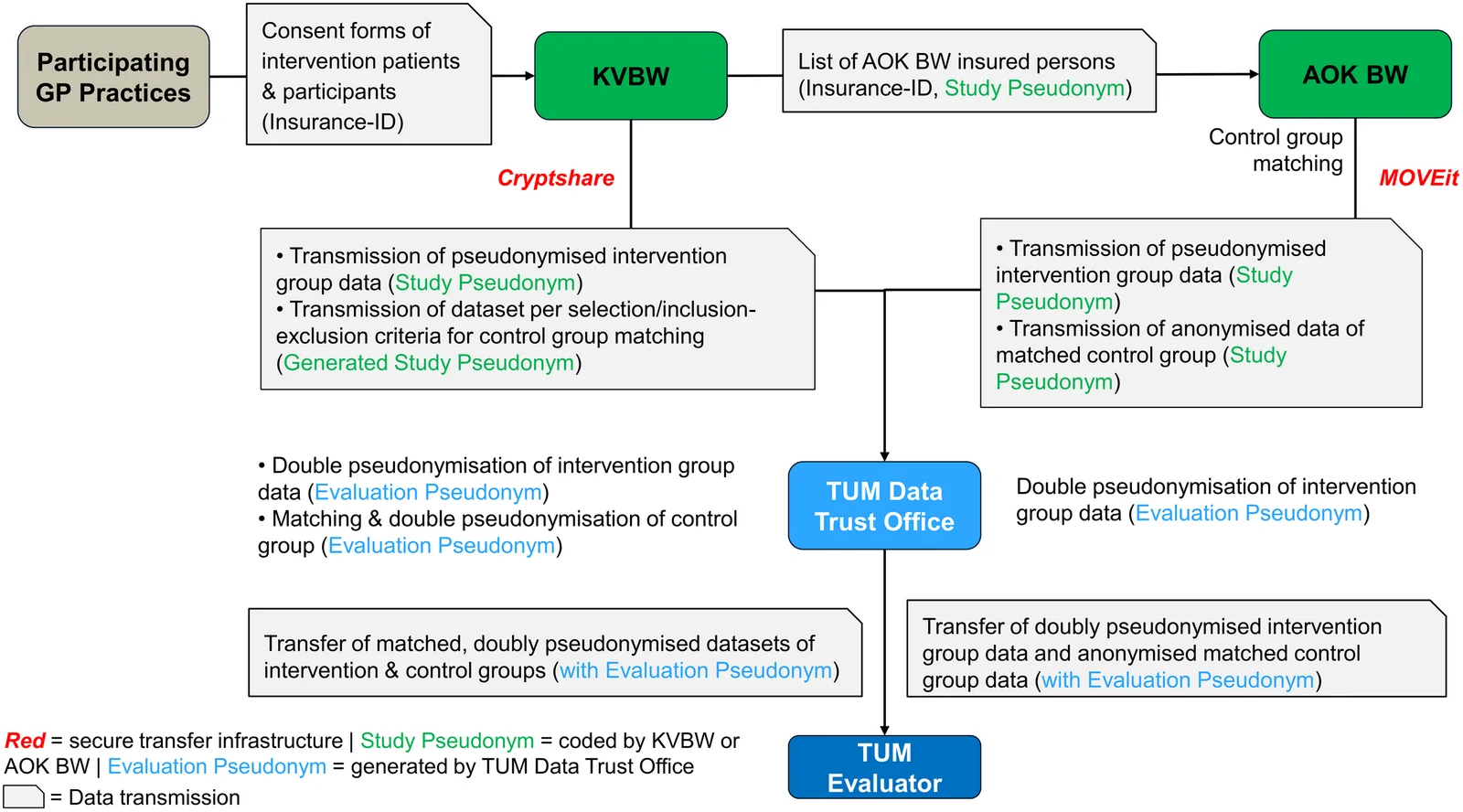
Data flow chart.

Data from both providers are pseudonymized prior to transfer. A double-pseudonymization procedure is applied by the TUM data trust office, replacing study pseudonyms with evaluation pseudonyms to prevent re-identification. The corresponding linkage key is stored under trustee responsibility and is not accessible to the evaluators. Data transfer to the evaluator is performed via secure, encrypted channels and external encrypted storage media, with access restricted to authorized personnel. Fig 3 illustrates the data flow.

Data will be used during the active project phase (01 January 2025–30 June 2028). In addition, data access will be maintained for up to 18 months after the end of the project to allow for additional analyses in the context of the peer-review and publication process. Subsequently, datasets will be securely archived in encrypted form and stored for 10 years in accordance with Good Practice for Secondary Data Analysis guidelines, after which they will be permanently deleted [27].

For the PE, quantitative data from participating patients will be collected using paper-based questionnaires distributed by the PN and subsequently entered into a Research Electronic Data Capture (REDCap) database after completion. Alternatively, participants may complete the questionnaires directly via a secure link to the REDCap database used within the PRIMA study. All questionnaires are pseudonymized at the patient level using participant-generated pseudonyms; no directly identifiable personal or medical information is accessible to the study team. Information on the reliability and validity of the questionnaires used in the target population is provided in Table 3. Duplicate entries will be excluded. Participating PNs will receive standardized training on instructing participants in questionnaire completion. Activity documentation is likewise pseudonymized at the patient level using the same pseudonym for matching purposes. Quantitative data documented by the practice teams are recorded anonymously. Qualitative data will be collected through individual and group interviews conducted either in person or via Zoom. Interviews will be audio-recorded and transcribed verbatim. All interviewers will receive training prior to data collection. Transcripts will be pseudonymized, and all identifying information will be removed during the pseudonymization process. Audio recordings will be retained until completion of the analyses to allow clarification of potential transcription uncertainties. All transcripts will be reviewed by the respective interviewers for accuracy. In addition, qualitative activity documentations will be collected from the practice teams for three selected workdays. Participants will create these themselves in the form of written documents or audio files, and submit them to the scientists from the Institute of General Practice and Interprofessional Care, Tuebingen (IAIV). All qualitative activity logs will be transcribed and pseudonymized if necessary, using the same procedure as for the interviews.

All study data will be stored for 10 years after completion of the analyses in accordance with international standards for good scientific practice. Access to personal information will be restricted to authorized study personnel only.

### Safety considerations

No direct medical harms are expected from participation in the PRIMA trial, as the intervention does not involve pharmacological or invasive procedures. Adverse events are not expected to occur in a systematic clinical sense and will therefore not be collected using a structured adverse event reporting system. Potential risks are primarily organizational in nature and may include increased workload or temporary workflow disruptions within participating practices during implementation of the intervention. Participation in the PE is voluntary and not associated with any major risks or burdens. It is possible that participants may feel uncomfortable or burdened during the interviews. If this occurs, they may contact the study leadership. Participants are free to discontinue the interview at any time and to withdraw their consent to participate. If participants find it difficult to answer certain questions in the questionnaires or interviews, these may be skipped. The theoretical risk of data loss cannot be completely ruled out. Any relevant unintended effects reported by participating practices will be documented descriptively and considered in the PE. All identified unintended effects meeting relevance criteria for service delivery will be reported in study publications.

Protocol deviations are primarily relevant at practice level, as the intervention is implemented at cluster (practice) level. Deviations may include partial or delayed implementation of intervention components, such as incomplete integration of the PN. These deviations will be documented descriptively within the PE but will not affect inclusion of practices or patients in the primary effectiveness analyses. At patient level, protocol deviations are not applicable, as outcomes are assessed using routinely collected data independent of patient-level intervention adherence.

### Statistical analysis

#### Matching.

The analysis population comprises patients enrolled in the intervention practices and matched CGs identified from routine data of the Association of SHI Physicians Baden-Württemberg (KVBW) and AOK BW, respectively.

First, matching variables associated with treatment allocation and study outcomes will be defined based on a literature review and expert consultation [28]. Patient-level variables will include sociodemographic and clinical characteristics such as age, sex, and the number of ASE in the pre-observation period. Practice-level variables will include structural characteristics such as practice type and size, approximated by level of care provision or the number of physicians per practice. Additionally, regional indicators such as the density of doctors or the average distance to the nearest GP are considered.

Second, suitable matching methods will be evaluated regarding methodological appropriateness and feasibility using routine data. Given the multilevel structure of the intervention, several approaches will be considered, including Propensity Score Matching, Coarsened Exact Matching, Optimal Full Matching, and cluster-based matching approaches for observational studies [29–32].

Matching will be conducted separately for the two data sources: KV data will be matched at TUM, while AOK BW data will be matched directly by AOK BW. The resulting matched datasets will be assessed for covariate balance and comparability between intervention and CG [33]. The preferred matching approach will be the method achieving the best balance across predefined matching variables and thereby minimizing selection bias.

#### Effectiveness evaluation.

The primary endpoint (number of ASE after 12 months) will be analyzed according to the ITT principle. The ITT population of the IG comprises all patients enrolled in the NCM by the end of the nine-month recruitment period, receiving at least 12 months of care. The smallest unit of analysis is the individual patient, as outcomes are assessed at patient level. In a baseline analysis, sociodemographic and clinical characteristics of the IG will be compared with those of the matched CG to assess the effectiveness of the matching procedure and identify potential determinants of the study endpoints [34,35]. The significance of the differences will be tested using linear mixed models for continuous variables with normal distribution, logistic models for binary variables, and the chi-square test and Fisher’s test for categorical variables. Subsequently, the rate of ASE in the IG and CG will be analyzed using multi-level regression models with patients nested within practices to account for the hierarchical structure of the data [36]. As a sensitivity analysis, models with cluster-robust standard errors at the practice level will be estimated. Relevant covariates identified in the initial analyses will be included in adjusted regression models [37].

Continuous secondary endpoints, including healthcare costs, will be analyzed using adjusted mixed linear models. Mortality will be assessed using survival analyses, while binary outcomes will be analyzed using generalized linear mixed models. Covariates will be selected analogously to the primary analysis. Extensive sensitivity analyses are planned to assess the robustness of the evaluation parameters. The choice between matched datasets derived from KVBW or AOK-BW routine data will depend on the respective endpoint as specified in Table 2. Statistical significance will be defined as a two-sided p-value <0.05. Where relevant, 95% confidence intervals are provided.

### Health economic evaluation

The health economic evaluation will first assess the costs of ambulatory care within the IG from an ambulatory care payer perspective and relate these costs to the number of ASE. Results will be presented as incremental cost-effectiveness ratios (ICERs). Costs considered will include both project-specific costs (e.g., employment of the PNs and the PRIMA reimbursement model) and ambulatory healthcare expenditures reimbursed by SHI funds. Resource utilization will be derived from routine data and valued using standardized unit costs. Sensitivity analyses will be conducted to assess the robustness of the results, while statistical uncertainty of the ICERs will be estimated using cost-effectiveness acceptability curves. In addition to the SHI physician perspective, a separate analysis will be conducted from the perspective of participating general practices. This analysis will compare implementation-related costs (e.g., employment of PNs) with additional revenues generated through newly established reimbursement codes in order to assess the economic feasibility of the NCM.

For the population insured by AOK BW, total healthcare costs from the sickness fund perspective will be analyzed. In addition to ambulatory care costs, this analysis will include further sectors of care, such as inpatient care, medical aids and appliances, pharmaceuticals, rehabilitation services, and sickness benefits, where available in the routine data. These costs will subsequently be related to the number of ASE using ICERs. Additional sensitivity analyses and cost-effectiveness acceptability curves will be used to assess uncertainty in the economic evaluation.

All statistical analyses will be conducted in R (latest version; R Foundation for Statistical Computing) using RStudio or comparable tools, according to predefined standard operating procedures (SOPs).

### Process evaluation

Questionnaire-based data and activity logs will be analyzed descriptively. Inferential statistical analyses will be conducted as exploratory secondary analyses when justified by hypotheses emerging from the integration of qualitative data and/or exploratory quantitative analyses. Qualitative case study data will be analyzed using reflexive thematic analysis as described by Braun and Clarke [38,39]. Following a predominantly inductive and interpretive approach, data will be coded and iteratively analyzed to identify patterns of meaning across cases along a six-phase process described by Braun and Clarke. Theme development will be guided by researcher reflexivity and ongoing discussion within the research team comprising nursing researchers, an APN, GPs and a social scientist.

### Subgroup analysis

In addition, predefined subgroup analyses will be performed to investigate the effects of the NCM in different or particularly affected patient populations. This includes separate analyses of patients who, at the time of study inclusion, were in an unstable phase of a chronic condition or in a pre-deterioration phase with a high risk of decompensation. If the sample size is sufficiently large, further subgroup analyses will be conducted, including stratification by age, sex, and presence of chronic conditions.

The statistical approach for subgroup analyses will initially be descriptive and subsequently, in line with the main effectiveness and health economic analyses, based on regression models for the primary endpoint as well as selected secondary endpoints. For the PE there are no pre-specified subgroup analysis planned as results of data integration will lead further analysis.

### Missing data

Missing data are expected to be minimal, as outcome data are derived from routinely collected administrative healthcare databases (SHI and KV data). Nevertheless, incomplete follow-up may occur due to changes in insurance status, migration outside the data capture area, or death. Such missingness will primarily result in right-censoring of observation periods. Analyses will account for this using appropriate model-based approaches inherent to survival and mixed-effects regression models, which allow inclusion of all available observations under a missing-at-random assumption conditional on observed covariates. No imputation will be performed for fully missing outcome periods; however, sensitivity analyses will be conducted to assess the robustness of results to potential informative censoring. For the quantitative part of the PE, multiple imputation approaches will be applied if needed.

### Oversight and monitoring

The KVBW holds overall responsibility as consortium lead and oversees project management as well as contractual coordination. Monitoring measures include regular oversight of study progress through milestone-based reporting to the sponsor on recruitment and billing figures, risk-based monitoring to identify critical study sites or processes, and regular consortium meetings to evaluate overall project progress. IGES supports the implementation process, contributes to the development of the concept of care, ensures peer communication and feedback throughout the intervention, monitors intervention activities via the work diary and provides care-related expertise. IAIV is responsible for PE. Technical University of Munich (TUM) conducts effectiveness and health economic evaluations. The Central Institute for SHI Physician Care (Zentralinstitut kassenärztliche Versorgung, Zi) supports the implementation and use of SmED.

Given the low-risk, non-pharmacological nature of the PRIMA study and the use of routinely collected administrative healthcare data, no independent Data Monitoring Committee (DMC) has been established.

Study oversight is provided by the Trial Steering Committee, which is responsible for monitoring overall study conduct, data quality, and adherence to the study protocol. As no investigational medicinal product or intervention with expected safety concerns is involved, formal interim safety monitoring by an independent DMC is not considered necessary. Given the pragmatic, routine data–based nature of the PRIMA study, no formal external audit program of trial conduct is planned. Data quality and study conduct are ensured through routine data validation procedures implemented by SHI providers and the Association of Statutory Health Insurance Physicians (KV). In addition, ongoing project monitoring is conducted by the consortium lead to ensure adherence to the study protocol and data completeness.

### Ethical considerations and declarations

The study received approval from the Ethics Committee of the State Medical Association of Baden-Württemberg, Germany (F-2025–129) and the Ethics Committee of the Medical Faculty at the University Tuebingen (177/2025BO2).

## Discussion

### Expected results and limitations of the study design

The intervention is expected to improve the care for chronically ill patients treated by general practice teams in Germany. The reduction of ASE hereby serves as a proxy for the improvement of continuity of care and more proactive patient management by the PN, reflected in the primary endpoint of the study. Furthermore, it is expected that care delivery can be further enhanced through task-shifting, particularly by delegating selected clinical tasks from physicians to PNs. In addition, the stronger integration of non-physician healthcare professionals could contribute to changes in practice organization and help reduce physician workload through more structured care for patients with chronic conditions. On the patient side, the intervention aims at strengthening patient self-management and health literacy. Thereby a further workload reduction is expected. Putting this together, the PRIMA project will add evidence to the further development of general practice teams in Germany. In comparison to earlier projects like FAMOUS, an academic qualification of the PN is not necessary for participation. Thereby, PRIMA also adds value to the question of the role of nursing professionals in general practice in Germany in regard to the academic qualification level of the nurses as there is a relatively small academic nursing working force in Germany. The study may further contribute to the evidence base for implementing NCM in primary care in Germany and to ongoing discussions on the primary care reform. Findings could inform future reimbursement structures for multiprofessional care and support efforts to shift care from inpatient to ambulatory settings, thereby reducing pressure on emergency departments and hospitals.

However, several limitations should be acknowledged. Implementation of the intervention is likely to vary across participating practices, particularly regarding the roles and responsibilities assumed by PNs. Differences in practice size, team culture, and digital infrastructure may influence implementation processes and contribute to heterogeneity in intervention effects. In addition, adaptive implementation and learning effects over time are expected, as practices may locally modify intervention components and improve integration throughout the study period. Furthermore, training level and experience of the PN could influence the study outcomes.

Potential unintended effects should also be considered. The introduction of new professional roles and structured care processes may initially increase coordination demands within practice teams. On the patient level, uncertainty regarding the respective roles of nursing professionals and physicians may occur during early implementation phases. Furthermore, improved access and proactive care management could temporarily increase consultation rates and affect economic evaluation.

Voluntary participation may also introduce selection bias, as practices with a stronger interest in innovation and team-based care may be more likely to participate, which should be considered when interpreting the generalizability of the findings. In addition, the 12-month follow-up period may be insufficient to detect longer-term effects, particularly regarding healthcare utilization and costs. Potential drop-outs at both practice and patient level, including staff turnover and loss to follow-up, may affect study implementation and outcome assessment. These risks were considered in the study planning and sample size calculation. The use of separate routine data sources from AOK BW and KVBW, which are not linked at the individual level, limits the possibility of fully integrated analyses. Moreover, the validity of the matched CG depends on the availability of routine data variables and the applied matching procedure, which may not fully account for residual differences between groups. However, matching considerably improves the comparability of both groups by reducing observable baseline differences.

Despite these limitations, the study has considerable transfer potential. Owing to the relatively uniform structure of the German statutory healthcare system, the findings are expected to be highly transferable within Germany. The results may also be relevant internationally, as many healthcare systems face similar challenges related to multimorbidity, workforce shortages, and the growing need to strengthen primary care. However, transferability to other countries will depend on the organization of primary care, financing structures, and the regulatory scope of practice for nursing professionals. The accompanying PE will therefore be essential for interpreting implementation and effectiveness across different contexts. In this regard, PRIMA may serve as a blueprint for future initiatives aimed at strengthening multiprofessional and team-based primary care models.

### Dissemination plans

No interim analyses or stopping guidelines are planned for this trial. Results will be communicated in form of scientific publications including reporting in the DRKS. The results will be further disseminated in a target group–specific manner to study participants and the wider scientific community. In accordance with funding requirements of the Innovation Fund, a final study report and results publication will be produced at the end of the project.

Any substantial amendments to the study protocol will be documented and, if required, submitted for approval to the relevant ethics committee and funding body. In case of premature termination of the study, data collected up to that point will be retained and analyzed in accordance with applicable data protection regulations and study objectives. Termination decisions will be documented and reported transparently.

## Supporting information

S1 File SPIRIT checklist. (PDF)

S2 File TREND checklist. (PDF)

## Abbreviations

AOK BW: Allgemeine Ortskrankenkasse Baden-Württemberg (statutory health insurance fund)
APN: Advanced Practice Nurse
ASE: Ambulatory care-sensitive emergency cases
CFIR: Consolidated Framework for Implementation Research
CCM: Chronic Care Model
CG: Control group
DMC: Data Monitoring Committee
EBM: Einheitlicher Bewertungsmaßstab (German uniform value scale for outpatient care billing)
GP: General practitioner
IAIV: Institute of General Practice and Interprofessional Care
ICC: Intra-cluster correlation coefficient
ICER: Incremental cost-effectiveness ratio
IG: Intervention group
IGES: Institute for Health and Social Research; ITT: Intention-to-treat
KV: Kassenärztliche Vereinigung (Association of Statutory Health Insurance Physicians)
KVBW: Kassenärztliche Vereinigung Baden-Württemberg
NäPA: Nichtärztliche Praxisassistenz (Non-medical Practice Assistant)
NCM: New care model
PE: Process evaluation;
PN: PRIMA nurse
PRIMA: Transforming GP Practices into Multiprofessional Primary Care Centers: The Role of Nursing Professionals (Project title)
REDCap: Research Electronic Data Capture
SHI: Statutory health insurance
SmED: Strukturierte medizinische Ersteinschätzung in Deutschland (structured medical initial assessment tool)
SOP: Standard operating procedure; TUM: Technical University of Munich
VERAH: Versorgungsassistentin in der Hausarztpraxis (GP-Practice Assistant)
Zi: Zentralinstitut für die kassenärztliche Versorgung (Central Institute for Statutory Health Insurance Physicians)
